# President’s Address before Atlanta Medical Society*Inaugural Address delivered January 1, 1895.

**Published:** 1895-02

**Authors:** C. D. Hurt

**Affiliations:** Atlanta, Ga.


### PRESIDENT'S ADDRESS BEFORE ATLANTA MEDICAL SOCIETY.*

BY C. D. HURT, M. D., ATLANTA, GA.

GENTLEMEN:—In calling me to preside over your deliberations for the next twelve months, you have conferred upon me an honor which I greatly appreciate. I shall assume such a responsibility with some trepidation; yet, with a determination to put forth an energy, zeal and impartiality, which I trust will give satisfaction to one and all. You have had a number of worthy and competent men to assume these duties in the past, and I shall count myself fortunate if I may but emulate their noble examples. In endeavoring to do this, I shall not forget that our watch-word is "*Advance*."

Our science has accomplished much in this the nineteenth century, of which we ought to feel proud; and our work promises to culminate in the last decade in one grand invasion upon the myriads of bacillæ which have in former years held high carnival over the destruction of the human race. If you and I live to see the dawn of the twentieth century, we shall be able to receive its first rays as so many indices pointing us to higher and still greater attainments.

While Jenner, Pasteur, Koch, Behring and others have lifted us into new fields for rich harvest, they have left us to separate the tares before garnering the wheat. They have indicated to us the paths for profitable research. It is our duty and high privilege to delve with such problems until the mists shall have been cleared away and we shall be able to prove to the world ur absolute success in exterminating epidemics and contagions. Microscopy, bacteriology, and the germ-culture which familiarize us with the nature and habits of these vile intruders; asepsis, cleanliness of person and apparel, sterilization of food and drink as means of preventing contact with them; antiseptics; antitoxine, tuberculin, and the germicides in general, by which we destroy these bacillæ when they have found a lodgement about our bodies, are the fields which most engage us at present. Through the aid of these hygienic influences the surgeon has been enabled to get out of the quagmire of putrefac-

*Inaugural Address delivered January 1, 1895.

tive filth and invest his operating department with absolute neatness.

We, as Americans, have much at which to rejoice; for while our fathers went over and were taught from the great laboratories of our eastern progenitors, we have proven ourselves worthy students and co-laborers, and are to-day returning to them many gems of scientific discovery. American doctors are to-day keeping in the front rank and vying with their oriental brethren. The tidal wave of prosperous scientific growth is now flushing our land and country. Our schools are multiplying and grading their courses, thereby elevating the standard for the profession. From the American Medical Association emanates a light which is to-day casting a glow over the entire universe.

When we look at the history of our commonwealth we note with pride what she has contributed to our science. Here in the capital city, the legislature of Georgia has honored itself and all Georgians by perpetuating the name of Dr. Crawford W. Long, "the discoverer of anæsthesia." His portrait hangs in yonder hall, and every Georgian's heart throbs with love for his memory.

While we have accomplished much, there yet remains much to be done. In the past few week, after two or three years of persistent effort from some of our self-sacrificing doctors, our legislature has enacted a prohibition against quacks and charlatans. On account of such previously existing laws in other states, Georgia has until now been a dumping-ground, free to all impostors.

Following in the wake of this important step, the question of a State board of health confronts us. Already in thirty-seven of our States such a law is in operation; and Mr. Cleveland has wisely recommended to Congress the adoption of a national board. Our State, with its two and a half millions of people, with its immense wealth, with its length of sea coast, exposed to invasion by foreign epidemics, and with its reputation for keeping abreast with the times, cannot afford to be without such a law.

There is another matter to which I desire to call attention and in which I hope to see enlisted the active minds of the profession. While we are having laws in every State regulating the licensing of physicians and druggists, and raising the standard so to allow only competent men to participate in this work, there seems to be an evil which is still at liberty and greatly handicaps the progress of science. I allude, gentlemen, to the vendors of patent medicine and the speculating imitators of truly scientific and valuable pharmaceutic preparations. No sooner does some man after arduous and continued toil announce that he has evolved Tuberculin, Antitoxine, etc., than spurious and worthless imitations are placed on the market and bring discredit to that which might prove a great boon to suffering humanity. If we had a commission of competent men whose duty it should be to pass upon the genuineness of every preparation and attach thereto a certified copy of its formula, much harm would be averted and much good accomplished.

Why, if the workmen are required to be skilled and competent, does not our government say they shall have well-wrought instruments? Why, if the physician and druggist shall be so careful and held responsible for human life, should not the manufacturers of pharmaceutic remedies be equally bound?

If what I have said inspires you to make renewed effort, I shall feel that I have not spoken in vain. The science of medicine means study and the interchanging of observations and ideas. For this purpose has this society been organized; for this purpose do we meet here from time to time; to this end let us labor faithfully, and may the Great Master of Spirits guide us.

